# Community Structure and Diversity of Scolytinae in Two Mangrove Ecosystems of Western Mexico

**DOI:** 10.3390/insects17090922

**Published:** 2026-09-02

**Authors:** Julyanna Figueroa-Vázquez, Nestor Eduardo Fernández-Torres, Jesús Enrique Castrejón-Antonio, María Cruz Rivera-Rodríguez, Sergio Aguilar-Olguín, Daniel Alberto Montes-Galindo, Liliana Martínez-Venegas, Patricia Tamez-Guerra

**Affiliations:** 1 Faculty of Biological and Agricultural Sciences, University of Colima, Autopista Colima-Manzanillo km 40, La Estación, Tecomán C.P. 28930, Mexico; jfigueroa36@ucol.mx (J.F.-V.); nfernandez1@ucol.mx (N.E.F.-T.); mrivera4@ucol.mx (M.C.R.-R.); 2 Faculty of Marine Sciences, University of Colima, Carretera Manzanillo-Barra de Navidad km 19.5, El Naranjo, Manzanillo C.P. 28868, Mexico; saguilar14@ucol.mx; 3 Faculty of Chemical Sciences, University of Colima, Carretera Colima-Coquimatlán km 9, Coquimatlán C.P. 28400, Mexico; daniel_montes@ucol.mx; 4 Bachillerato 33, University of Colima, Av. Griselda Alvarez Ponce de León, S/N, Cuauhtémoc C.P. 28500, Mexico; liliana_martinez@ucol.mx; 5 Department of Microbiology and Immunology, Faculty of Biological Sciences, Autonomous University of Nuevo León, Av. Pedro de Alba S/N, Cd. Universitaria, San Nicolas de los Garza C.P. 66455, Mexico; patamez@hotmail.com

**Keywords:** ambrosia beetles, biodiversity, coastal wetlands, forest entomology, phytosanitary monitoring

## Abstract

Mangroves support a diverse insect fauna that plays essential roles in ecosystem functioning; however, the diversity of bark and ambrosia beetles (Curculionidae: Scolytinae) in the mangroves of the Mexican Pacific remains poorly understood. This study aimed to characterize the diversity and community structure of Scolytinae associated with two mangrove ecosystems in the state of Colima, western Mexico: Palo Verde Estuary and Juluapan Lagoon. Beetles were sampled using ethanol-baited traps (70% ethanol). A total of 1869 individuals belonging to 19 species and 10 genera were collected. *Hypothenemus* was the most species-rich genus, whereas *Xyleborus volvulus* was the dominant species. Five species are reported for the first time in Colima: *Ambrosiodmus obliquus*, *Corthylus comatus*, *Premnobius cavipennis*, *Hypothenemus dolosus*, and *Hypothenemus indigens*. Palo Verde Estuary showed higher beetle abundance, whereas Juluapan Lagoon exhibited greater species diversity and community evenness. Although the community shared several genera with mangrove ecosystems from other regions of Mexico and Brazil, it differed in species composition and dominance patterns. This study provides the first baseline on the Scolytinae fauna associated with the mangroves of Colima and establishes a reference for future ecological monitoring and phytosanitary surveillance programs.

## Simple Summary

Mangroves are coastal ecosystems that support a high diversity of organisms and provide essential ecological services. Among the insects inhabiting these environments are beetles of the subfamily Scolytinae (Coleoptera: Curculionidae), which contribute to wood decomposition and nutrient cycling, although some species may become important pests. Despite their ecological importance, little is known about these beetles in the mangroves of the Mexican Pacific. In this study, we assessed their diversity in two mangrove ecosystems in the state of Colima, western Mexico. We recorded 19 species belonging to 10 genera, including five species reported for the first time in the state. *Hypothenemus* was the most species-rich genus, whereas *Xyleborus volvulus* was the most abundant species. Although the two mangroves shared most of the recorded genera, they differed in species abundance and community structure. We did not detect *Coptoborus pseudotenuis*, a species considered a phytosanitary threat to Mexican mangroves. Our findings provide the first baseline information on the Scolytinae fauna associated with the mangroves of Colima and establish a reference for future studies on biodiversity, conservation, and phytosanitary monitoring.

## 1. Introduction

Mangroves are forested coastal ecosystems dominated by trees and shrubs adapted to saline or brackish environments, primarily in tropical and subtropical regions [1]. They are among the most productive ecosystems on Earth because of the wide range of ecosystem services they provide, including shoreline protection against erosion and extreme weather events, carbon sequestration, and the maintenance of biodiversity by serving as feeding, shelter and breeding habitats for numerous species of fish, crustaceans, mollusks, birds, reptiles, and mammals [2,3]. Mangroves also support a diverse arthropod fauna, particularly insects that play key ecological roles in herbivory, pollination, predation, and the decomposition of organic matter [4,5].

Among this insect fauna, bark and ambrosia beetles of the subfamily Scolytinae (Coleoptera: Curculionidae) are particularly noteworthy because of their wide distribution in forest ecosystems and the remarkable diversity of ecological strategies exhibited by their species [6]. Many species colonize dead wood or weakened plant tissues, promoting wood decomposition and the reincorporation of organic matter into the ecosystem, thereby contributing to nutrient cycling [7,8]. However, some species can become important forest pests by attacking living or stressed trees, causing significant ecological and economic damage [9,10]. In this context, in 2022 the Mexican National Forestry Commission (CONAFOR) and the Ministry of Environment and Natural Resources (SEMARNAT) issued an early warning and risk assessment for *Coptoborus pseudotenuis* Wood & Bright, 1992, a species considered a potential threat to mangrove ecosystems in southeastern Mexico, particularly black mangrove, *Avicennia germinans* (L.) L., stands. The assessment identified the states of Oaxaca, Chiapas, Veracruz, Tabasco, Campeche, and Baja California Sur as being at very high risk of invasion, whereas Colima was classified as having a moderate-to-high risk [11].

Approximately 846 species of Scolytinae have been recorded in Mexico across a wide range of natural ecosystems and agroecosystems [12,13,14,15]. However, knowledge of Scolytinae communities associated with mangrove ecosystems remains limited. To date, the principal studies have focused on mangroves in the states of Tabasco [16,17] and Tamaulipas [18,19]. Outside of Mexico, only one study has been published, focusing on the mangrove of Rio de Janeiro, Brazil [20]. In contrast, virtually no information is available on Scolytinae inhabiting mangroves along the Mexican Pacific coast. This knowledge gap is particularly relevant in the state of Colima, which, despite its relatively small territorial extent, contains approximately 3400 ha of mangrove forests distributed across its three coastal municipalities [21]. Furthermore, although the database compiled by Atkinson [22] lists 44 Scolytinae species belonging to 16 genera for Colima—including records generated by previous studies conducted by our research group [23,24]—none had previously been reported from mangrove ecosystems or other coastal wetlands.

Given this knowledge gap and the ecological and phytosanitary importance of Scolytinae associated with mangrove ecosystems, this study aimed to characterize the diversity and community structure of Scolytinae in two representative mangrove ecosystems of the state of Colima, western Mexico: Palo Verde Estuary and Juluapan Lagoon. Our findings provide the first inventory of Scolytinae for mangrove ecosystems in the central Mexican Pacific and establish a baseline for future ecological, biogeographical, and phytosanitary studies aimed at assessing changes in community composition and detecting the potential introduction or establishment of species of phytosanitary importance, including *C. pseudotenuis*.

## 2. Materials and Methods

### 2.1. Study Area

**Figure 1:**
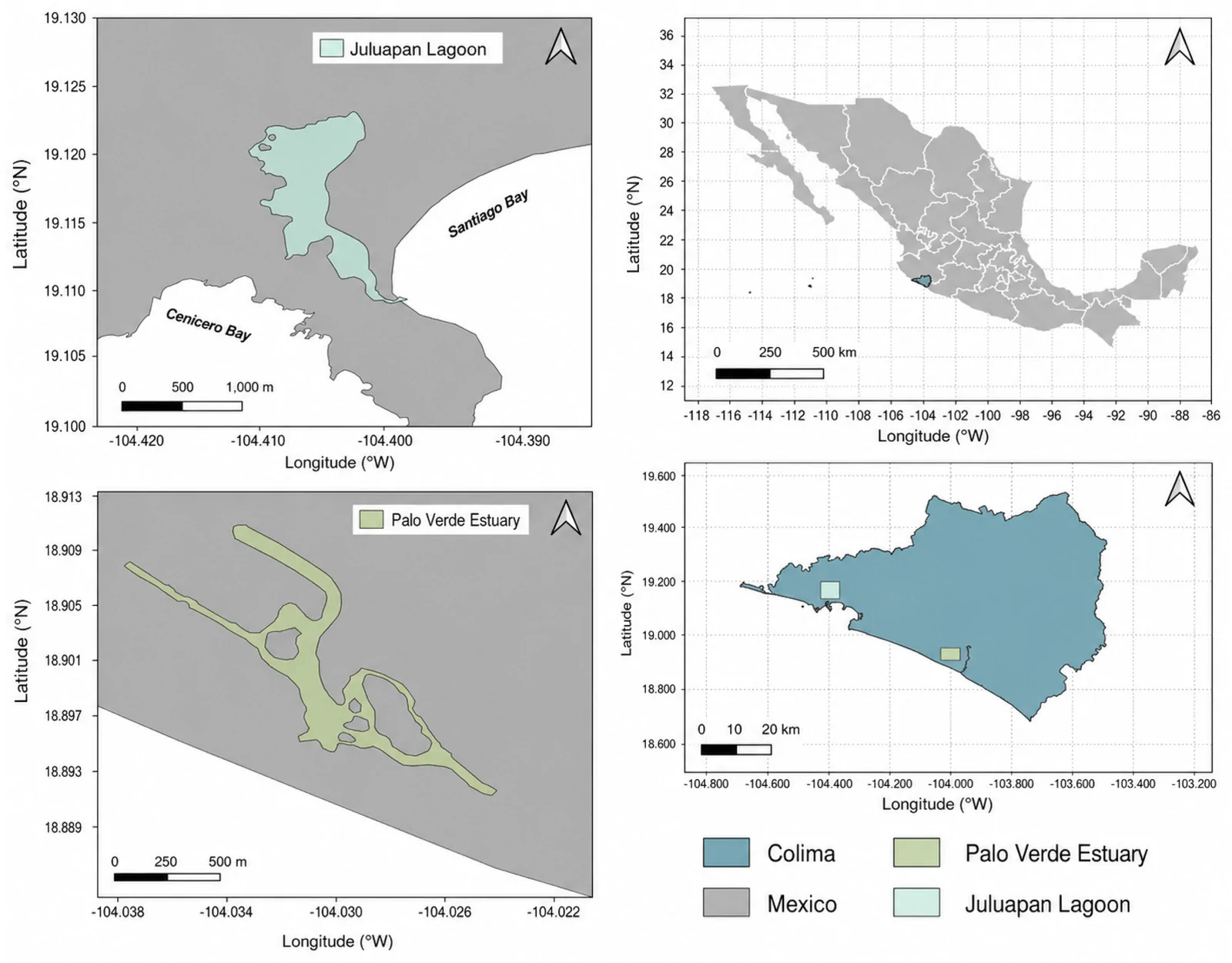
Location of the two mangrove study sites in Colima, Mexico.

**Figure 2:**
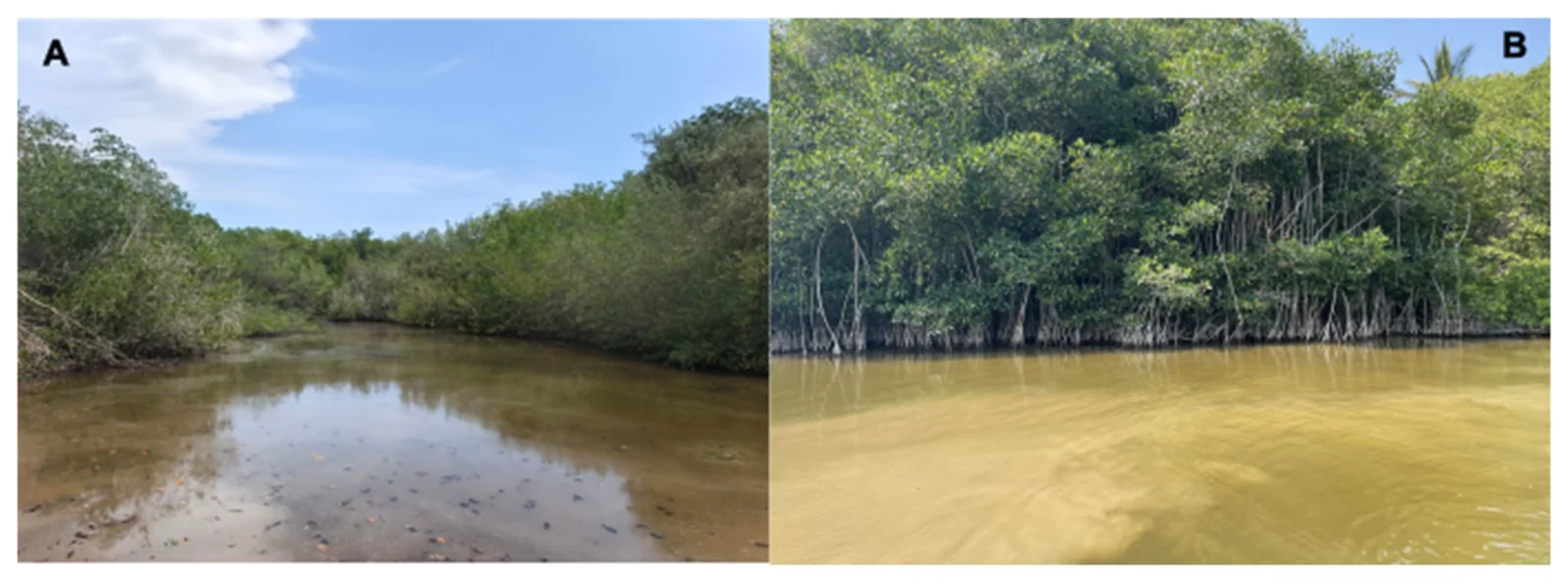
Juluapan Lagoon (**A**); Palo Verde Estuary (**B**).

The study was conducted in two mangrove ecosystems in the state of Colima, western Mexico: Palo Verde Estuary (PVE), located in the municipality of Armería (18°53′50″ N, 104°02′04″ W), and Juluapan Lagoon (JL), located in the municipality of Manzanillo (19°06′16″ N, 104°23′56″ W) (Figure 1). These sites were selected because they represent well-established mangrove ecosystems within the coastal zone of Colima and have been previously characterized in terms of their mangrove structure [25,26]. PVE forms part of the Cuyutlán Lagoon system, one of the major coastal lagoon systems of the state, whereas JL is a distinct coastal lagoon associated with Bahía de Santiago. At PVE, beetle sampling was conducted from January to March 2024, when water levels allowed access to the study area. This ecosystem is characterized as a riverine mangrove dominated by *Rhizophora mangle* L. (red mangrove) and *Laguncularia racemosa* (L.) C.F.Gaertn. (white mangrove) (Figure 2). At JL, sampling was carried out in areas dominated by *L. racemosa* during March, May, June, August, October, and December 2025, corresponding to the months in which access to the study area and field monitoring were possible.

### 2.2. Insect Sampling and Taxonomic Identification

**Figure 3:**
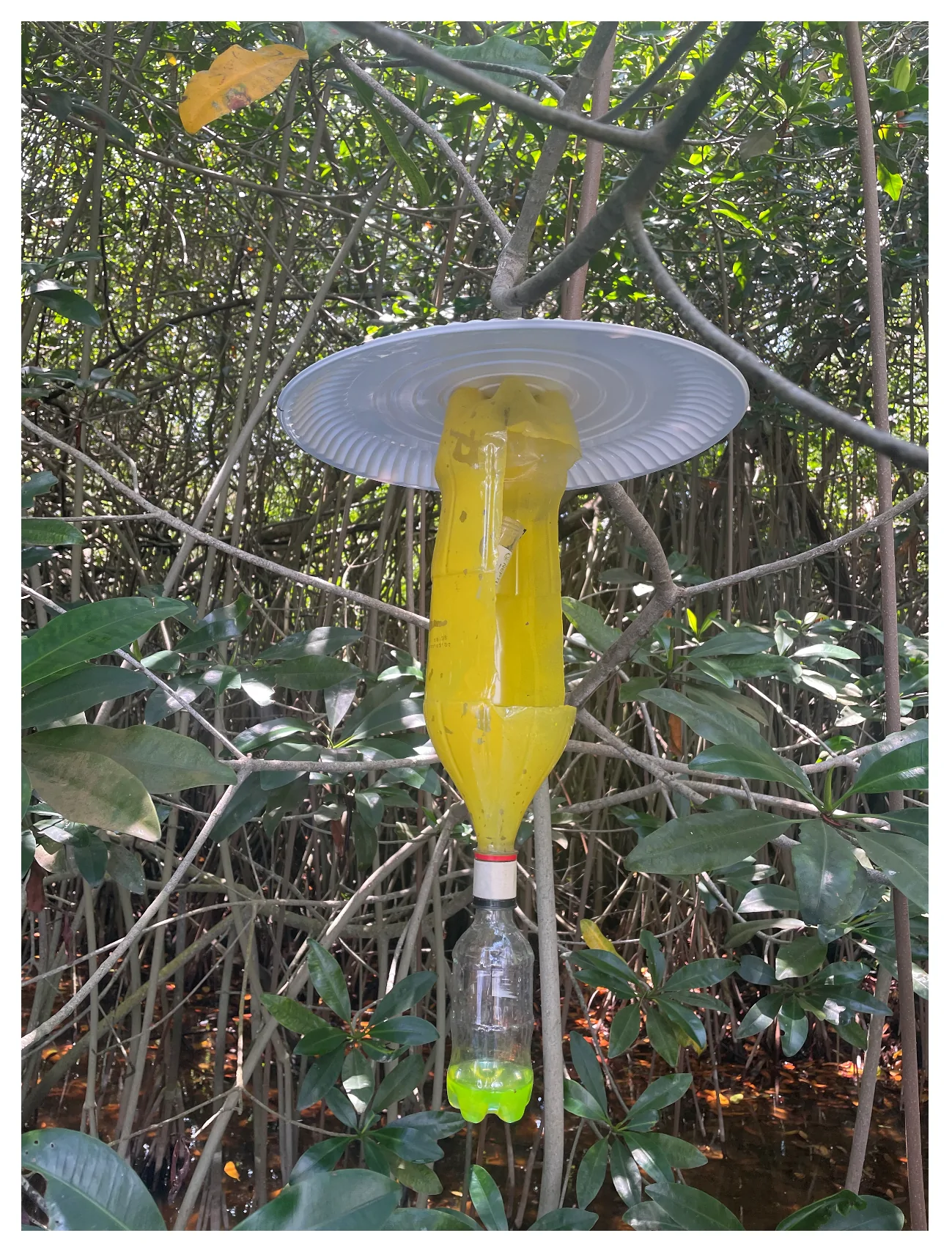
Ethanol-baited flight-intercept trap used for capturing ambrosia beetles.

Beetles were sampled using flight-intercept traps baited with 70% ethanol and fitted with collection bottles containing automotive antifreeze (Super Tech^®^, Ciudad de México, México) as a killing and preservative solution [27] (Figure 3).

Ten traps were installed at each location in a linear arrangement, at a height of 1.5 m above ground and spaced 100–150 m apart. Traps were inspected every 15 days throughout the study period. Collected specimens were transported to the laboratory, where they were transferred to 70% ethanol for preservation and identified using the taxonomic keys of Wood [28], Wood and Bright [29], and Atkinson [30]. Voucher specimens were subsequently deposited in the insect collection of the Faculty of Biological and Agricultural Sciences, University of Colima, Colima, Mexico.

### 2.3. Data Analysis

Species diversity was evaluated and compared among sampling months and between the two study sites using the Shannon–Wiener diversity index (H′), Margalef’s richness index (Dmg), Pielou’s evenness index (J), and Simpson’s dominance index (Ds). These indices were calculated from the abundance of individuals recorded for each species at each sampling period and study site. All analyses were performed using PAST (PAleontological Statistics version 5.3) software [31].

## 3. Results

A total of 1874 individuals belonging to 19 species and 10 genera of the subfamily Scolytinae were collected across the two study sites (Table 1). *Hypothenemus* was the most species-rich genus, comprising nine species and accounting for nearly 30% of the total species richness recorded. Within this genus, *Hypothenemus birmanus* was the most abundant species, representing approximately 47% of all *Hypothenemus* individuals collected. In contrast, the genus *Xyleborus* was represented by only two species, *Xyleborus volvulus* and *Xyleborus palatus*, which together accounted for more than 60% of the total number of individuals collected. *Xyleborus volvulus* was the dominant species at both study sites, with 1030 individuals, representing approximately 55% of the total abundance recorded during the study. Species such as *Corthylocurus* sp., *Corthylus comatus*, *Hylocurus elegans*, *Hylocurus inaequalis*, *Xylosandrus curtulus*, and *Monarthrum* sp. were represented by only a few individuals and were therefore considered rare within the sampled assemblage. Five species are reported for the first time from the state of Colima: *Ambrosiodmus obliquus*, *C. comatus*, *Premnobius cavipennis*, *Hypothenemus dolosus* and *Hypothenemus indigens*.

The two study sites differed in beetle abundance and species composition (Table 2). A total of 1180 individuals were collected at PVE, compared with 694 individuals at JL, despite the shorter sampling period at the former site. Genus richness was identical at both sites, with eight genera recorded in each. Species richness differed slightly, with 11 species recorded at PVE and 13 at JL. *Xyleborus volvulus* was the dominant species at both sites; however, its abundance was substantially higher at PVE (840 individuals) than at JL (190 individuals). Six species were recorded exclusively at PVE: *A. obliquus*, *Corthylus comatus*, *H. dolosus*, *H. indigens*, *Monarthrum* sp., and *X. curtulus*. Conversely, *Corthylocurus* sp., *H. elegans*, *H. inaequalis* and *Hypothenemus columbi* were found exclusively at JL.

The diversity indices further described variation in community structure between the two study sites. The Shannon–Wiener diversity index (H′) was higher at JL (2.02) than at PVE (1.08). Similarly, Margalef’s richness index (Dmg) was higher at JL (1.83) than at PVE (1.41). Pielou’s evenness index (J) also showed greater community evenness at JL (0.79) than at PVE (0.45), indicating a more uniform distribution of individuals among species. Conversely, Simpson’s dominance index (Ds) was substantially higher at PVE (0.53) than at JL (0.16), reflecting the strong numerical dominance of *X. volvulus* at this site.

Temporal variation in beetle abundance differed between the two study sites (Table 2). At PVE, the highest abundances were recorded during the first months of the sampling period (January–March). In JL, abundance fluctuated throughout the sampling period, reaching a peak in May (272 individuals) and declining markedly toward December, when only 10 individuals were collected.

Temporal changes in community structure were reflected by the diversity indices (Table 3). During January and February, diversity was low (H′ = 0.93 and 0.88, respectively), accompanied by low evenness (J = 0.42 and 0.40) and high dominance (Ds = 0.59 and 0.61). In March, diversity differed markedly between the two study sites. At PVE, diversity was moderate (H′ = 1.22, J = 0.58, Ds = 0.42), whereas JL exhibited the highest diversity recorded during the study (H′ = 2.11, J = 0.85, Ds = 0.15). During the subsequent sampling months at JL, diversity and evenness remained high, while dominance remained low, indicating a relatively stable community structure. By December, species richness declined to four species and diversity decreased to H′ = 1.23, although evenness remained relatively high (J = 0.89).

## 4. Discussion

The present study represents the first inventory of Scolytinae beetles associated with mangrove ecosystems in the state of Colima and expands the known fauna of the state through the first records of five species: *A. obliquus*, *C. comatus*, *P. cavipennis*, *H. dolosus* and *H. indigens*. These findings demonstrate that even in relatively well-studied states such as Colima, surveys conducted in poorly explored ecosystems continue to provide valuable information on the distribution of this group of beetles. Moreover, this study constitutes the first assessment of the Scolytinae fauna associated with mangroves along the central Mexican Pacific coast and complements previous inventories conducted in Tabasco [16,17] and Tamaulipas [18,19]. Species richness recorded in Colima was lower than that reported for the mangroves of Tabasco, where between 25 and 35 Scolytinae species have been documented [16,17], but was comparable to that observed in Tamaulipas, where up to 11 species have been recorded [19]. These differences may be associated with variation in mangrove area, structural complexity, and floristic composition [32,33], factors that strongly influence the availability of host resources and ecological niches for scolytine beetles [29,34,35,36].

The two mangrove ecosystems also differed in community structure. Juluapan Lagoon exhibited higher diversity and evenness, indicating a more even distribution of individuals among species. In contrast, PVE was characterized by the pronounced dominance of *X. volvulus*, resulting in lower evenness and higher dominance values. Although both sites are subject to anthropogenic influence, they differ in the surrounding landscape. Juluapan Lagoon is embedded within a predominantly urban matrix, whereas PVE is mainly surrounded by rural settlements and agricultural areas dominated by lime (*Citrus aurantiifolia* (Christm.) Swingle), papaya (*Carica papaya* L.), and coconut (*Cocos nucifera* L.) crops. These contrasting landscape settings suggest that local habitat heterogeneity and the availability of woody resources may contribute to shaping scolytine assemblages. However, this hypothesis should be evaluated through studies specifically designed to examine the relationships between habitat characteristics and Scolytinae community composition.

The most consistent pattern among the mangrove communities compared in this study was the high species richness of the genus *Hypothenemus*. In Colima, this genus accounted for the highest number of species, a pattern also reported from the mangroves of Tabasco, Tamaulipas, and Rio de Janeiro [16,17,19,20]. This recurring pattern is consistent with the remarkable ecological versatility of *Hypothenemus*, whose species exploit a wide variety of plant resources and exhibit diverse feeding habits, including herbivory, spermatophagy, myelophagy and xylomycetophagy. The combination of these ecological strategies enables *Hypothenemus* to occupy a broad range of ecological niches, making it one of the most species-rich and successful lineages within the subfamily Scolytinae [6,35,37].

Patterns of species dominance differed among the mangrove ecosystems compared. *X. volvulus* accounted for more than half of all individuals collected, mirroring the pattern reported for the mangroves of Tabasco, where this species was also clearly dominant [16,17]. In contrast, mangroves in Rio de Janeiro were dominated by *Xyleborus affinis* [20], whereas *Xyleborus bispinatus* was the only species of the genus recorded in Tamaulipas [19]. These differences suggest that, although *Xyleborus* is a recurrent component of mangrove-associated Scolytinae communities, the dominant species varies among regions, likely reflecting differences in vegetation composition, environmental conditions, and the availability of suitable host resources.

The high abundance of *X. volvulus* can be explained, at least in part, by its biological characteristics. Like other ambrosia beetles, it maintains a mutualistic association with symbiotic fungi that are cultivated within galleries excavated in the wood and serve as the primary food source for both larvae and adults [10]. This symbiosis enables the species to exploit a wide range of host plants and facilitates the colonization of stressed, dying, or decaying woody tissues [8,38,39]. The continuous production of dead branches and woody debris characteristic of mangrove ecosystems provides a persistent supply of suitable breeding substrates, likely favoring the establishment and proliferation of generalist ambrosia beetles such as *X. volvulus*.

Genus composition also revealed a common core among the mangrove ecosystems examined. *Cryptocarenus*, *Corthylus*, and *Premnobius* were recorded in Colima, Tabasco, Tamaulipas, and Brazil [16,17,19,20], suggesting that these genera represent characteristic components of mangrove-associated Scolytinae communities. Species composition, however, differed among regions. Whereas the mangroves of Tabasco and Tamaulipas were represented primarily by *Corthylus papulans*, *Corthylus minutissimus*, and *Cryptocarenus seriatus*, those of Colima harbored *C. comatus*, *Cryptocarenus heveae*, and *P. cavipennis*, thereby expanding current knowledge of the species composition of these genera in the mangroves of the Mexican Pacific.

The records of *H. elegans* and *H. inaequalis* are particularly noteworthy. Although both species had previously been reported from the state of Colima [22], neither had been documented from mangrove ecosystems. Their absence from published inventories of mangroves in Tabasco, Tamaulipas, and Rio de Janeiro [16,17,19,20] suggests that mangroves along the Mexican Pacific coast may harbor faunal elements that differ from those reported in other regions. Determining whether this pattern reflects biogeographic differences, variation in the surrounding vegetation, or differences in sampling effort will require additional surveys across mangrove ecosystems along the Pacific coast of Mexico.

The absence of *C. pseudotenuis* throughout the study period is an important finding from a phytosanitary perspective, given that this species has been identified as a potential threat to mangrove ecosystems in southeastern Mexico, particularly in stands of *A. germinans* [11]. Although this result only indicates that the species was not detected at the sampled sites during the study period, its absence may be related to differences in regional distribution [28], host availability, vegetation composition, or local environmental conditions. The concentration of previous records and phytosanitary concern in southeastern Mexico may also suggest a regional component to its occurrence; however, the available information is insufficient to determine whether these factors explain its non-detection in Colima. Targeted surveys involving potential host trees and additional mangrove sites, together with sampling across different seasons, would be necessary to evaluate this hypothesis. Nevertheless, the inventory generated here provides an important baseline for monitoring Scolytinae communities in the mangroves of Colima and for detecting any future introduction or establishment of *C. pseudotenuis* or other species of phytosanitary importance. Long-term monitoring may also facilitate the detection of changes associated with environmental disturbances, urbanization, hydrological alterations, and restoration programs.

Overall, the results demonstrate that the mangroves of Colima harbor a Scolytinae community that shares a common generic core with mangroves from other regions of Mexico and Brazil but differs in species composition and dominance structure. These patterns likely reflect the combined influence of environmental conditions, floristic composition, host availability, and regional biogeographic history on the assembly of Scolytinae communities [6,34,35,36].

## 5. Conclusions

Five species of Scolytinae beetles, *A. obliquus*, *C. comatus*, *P. cavipennis*, *H. dolosus*, and *H. indigens*, were first described and associated with mangrove ecosystems in the state of Colima, Mexico. Species from Colima differ in species composition and dominance structure compared with other Mexican states. Expanding comparable studies across mangroves of the Mexican Pacific will improve our understanding of the ecological and biogeographic processes shaping these assemblages while providing a broader context for evaluating regional biodiversity patterns.

## Figures and Tables

**Table 1:** Species of the subfamily Scolytinae collected from two mangrove ecosystems in the state of Colima, Mexico.

Species	Estero Palo Verde (EPV)	Laguna de Juluapan (LJ)	Total
*Ambrosiodmus obliquus* Blackman, 1928	46	0	46
*Corthylus comatus* Blandford, 1904	2	0	2
*Corthylocurus* sp.	0	1	1
*Cryptocarenus heveae* Wood & Bright, 1992	17	51	68
*Hylocurus elegans* Eichhoff, 1878	0	2	2
*Hylocurus inaequalis* Wood, 1956	0	3	3
*Hypothenemus birmanus* Wood & Bright, 1992	127	141	268
*Hypothenemus columbi* Hopkins, 1915	0	12	12
*Hypothenemus dolosus* Wood, 1974	25	0	25
*Hypothenemus eruditus* (Westwood, 1834)	0	12	12
*Hypothenemus indigens* Wood, 1974	87	0	87
*Hypothenemus javanus* Wood & Bright, 1992	0	34	34
*Hypothenemus rotundicollis* Wood & Bright, 1992	0	53	53
*Hypothenemus squamosus* Wood & Bright, 1992	6	72	78
*Monarthrum* sp.	2	0	2
*Premnobius cavipennis* Wood & Bright, 1992	27	19	46
*Xyleborus volvulus* (Fabricius, J.C., 1794)	840	190	1030
*Xyleborus palatus* Wood, 1974	0	104	104
*Xylosandrus curtulus* Wood & Bright, 1992	1	0	1
Total	1180	694	1874

**Table 2:** Temporal distribution of Scolytinae species collected from two mangrove ecosystems in the state of Colima, Mexico.

Species	PVE				JL						
	Jan	Feb	Mar		Mar	May	Jun	Aug	Oct	Dec	Total
*Ambrosiodmus obliquus*	16	6	24		0	0	0	0	0	0	46
*Corthylus comatus*	1	0	1		0	0	0	0	0	0	2
*Corthylocurus* sp.	0	0	0		1	0	0	0	0	0	1
*Cryptocarenus heveae*	10	3	4		8	19	4	13	7	0	68
*Hylocurus elegans*	0	0	0		1	1	0	0	0	0	2
*Hylocurus inaequalis*	0	0	0		0	1	0	0	2	0	3
*Hypothenemus birmanus*	34	15	78		31	63	17	22	6	2	268
*Hypothenemus columbi*	0	0	0		11	0	0	0	0	1	12
*Hypothenemus dolosus*	4	0	21		0	0	0	0	0	0	25
*Hypothenemus eruditus*	0	0	0		10	1	0	1	0	0	12
*Hypothenemus indigens*	38	42	7		0	0	0	0	0	0	87
*Hypothenemus javanus*	0	0	0		15	9	1	6	3	0	34
*Hypothenemus rotundicollis*	0	0	0		5	35	5	6	2	0	53
*Hypothenemus squamosus*	0	6	0		17	17	14	16	2	6	78
*Monarthrum* sp.	1	1	0		0	0	0	0	0	0	2
*Premnobius cavipennis*	3	6	18		6	7	2	2	2	0	46
*Xyleborus volvulus*	333	266	241		47	60	28	26	28	1	1030
*Xyleborus palatus*	0	0	0		31	59	4	6	4	0	104
*Xylosandrus curtulus*	0	1	0		0	0	0	0	0	0	1
Total	440	346	394		183	272	75	98	56	10	1874

PVE, Palo Verde Estuary; LJ, Juluapan Lagoon.

**Table 3:** Monthly values of diversity indices for Scolytinae communities in two mangrove ecosystems in the state of Colima, Mexico.

Study Site	Sampling Month	N	S	H′	Dmg	J	Ds
PVE	January	440	9	0.93	1.31	0.42	0.59
	February	346	9	0.88	1.37	0.4	0.61
	March	394	8	1.22	1.17	0.59	0.42
JL	March	183	12	2.11	2.11	0.85	0.15
	May	272	11	1.9	1.78	0.79	0.18
	June	75	8	1.67	1.62	0.8	0.24
	August	98	9	1.89	1.75	0.86	0.18
	October	56	9	1.67	1.99	0.76	0.29
	December	10	4	1.09	1.3	0.79	0.42

PVE, Palo Verde Estuary; LJ, Juluapan Lagoon.

## Data Availability

The raw data supporting the conclusions of this article will be made available by the authors on request.

## References

[1] Kathiresan K., Rastogi R.P., Mahendra P., Dharmendra K.G. (2021). Mangroves: Types and Importance. Mangroves: Ecology, Biodiversity and Management.

[2] Alongi D.M., Alongi D.M. (2018). Mangrove Forests. Blue Carbon: Coastal Sequestration for Climate Change Mitigation.

[3] Rodríguez-Zúñiga M.T., Troche-Souza C., Vázquez-Lule A.D., Márquez-Mendoza J.D., Vázquez- Balderas B., Valderrama-Landeros L., Velázquez-Salazar S., Cruz-López M.I., Ressl R., Uribe-Martínez A. (2013). Manglares de México/Extensión, Distribución y Monitoreo.

[4] Falcón-Brindis A., Pérez-de la Cruz M., Mata-Zayas E.E., de la Cruz-Pérez A., Sánchez-Soto S., Burelo-Ramos C.M. (2018). Scolytinae y Platypodinae (Coleoptera: Curculionidae) de Tabasco, México. Acta Zool. Mex..

[5] Membere O., Bawo D.D.S., Onwuteaka J., Ugbomeh A.P., Nwosu O.R. (2021). Abundance and diversity of insects associated with *Rhizophora mangle* and *Avicennia germinans in* Bundu-Ama mangrove ecosystem of the Niger Delta, Nigeria. Sci. Afr..

[6] Kirkendall L.R., Biedermann P.H.W., Jordal B.H., Vega F.E., Hofstetter R.W. (2015). Evolution and Diversity of Bark and Ambrosia Beetles. Bark Beetles.

[7] Wood S.L. (1982). The Bark and Ambrosia Beetles of North and Central America (Coleoptera: Scolytidae), a Taxonomic Monograph. Gt. Basin Nat. Mem..

[8] Raffa K.F., Grégoire J.C., Lindgren B.S., Vega F.E., Hofstetter R.W. (2015). Natural History and Ecology of Bark Beetles. Bark Beetles.

[9] Peña J.E., Carrillo D., Duncan R.E., Capinera J.L., Brar G., McLean S., Arpaia M.L., Focht E., Smith J.A., Hughes M. (2012). Susceptibility of *Persea* spp. and Other Lauraceae to Attack by Redbay Ambrosia Beetle, *Xyleborus glabratus* (Coleoptera: Curculionidae: Scolytinae). Fla. Entomol..

[10] Hulcr J., Stelinski L.L. (2017). The Ambrosia Symbiosis: From Evolutionary Ecology to Practical Management. Annu. Rev. Entomol..

[11] Comisión Nacional Forestal (CONAFOR) Alerta Temprana y Evaluación de Riesgo para *Euplatypus parallelus* y *Coptoborus pseudotenuis*, Junio 2022. CONAFOR: Mexico, 2022. https://sivicoff.cnf.gob.mx/ContenidoPublico/01%20Avisos%20Publicos/mapas%20de%20alerta%20temprana/Euplatypus%20parallelus%20y%20Coptoborus%20%20pseudotenius%202022/Alerta%20Temprana%20y%20Evaluacion%20de%20Riesgo%20para%20Euplatypus%20parallelus%20y%20Coptoborus%20%20pseudotenuis%202022_.pdf.

[12] Quezada-García R., Jiménez-Sánchez E., Equihua-Martínez A., Padilla-Ramírez J. (2014). Escolitinos y platipodinos (Coleoptera: Curculionidae) atraídos a trampas tipo NTP-80 en Zapotitlán de las Salinas, Puebla, México. Acta Zool. Mex..

[13] Pérez-de la Cruz M., Zavaleta-Bastar P.G., De la Cruz-Pérez A. (2015). Aproximación al conocimiento de la diversidad de Scolytinae y Platypodinae (Coleoptera: Curculionidae) asociados a selvas de Tabasco, México. Entomotropica.

[14] Lázaro-Dzul M.O., Equihua-Martínez A., Romero-Nápoles J., González-Hernández H., Alvarado-Rosales D., CastañedaVildózola A., Suárez-Espinosa J. (2023). Population fluctuation of Scolytinae (Coleoptera: Curculionidae) in avocado (Persea americana Mill.) orchards in Michoacán, México. Rev. Colomb. Entomol..

[15] Castrejón-Antonio J.E., Ramos-Arias C.F., Sánchez-Rangel J.C. (2024). Escarabajos Scolytinae (Coleoptera: Curculionidae) asociados a una huerta de aguacate en el estado de Jalisco, México. AIA Av. Investig. Agropecu..

[16] Gerónimo-Torres J.D.C., Pérez-De La Cruz M., De La Cruz-Pérez A., Torres-De La Cruz M. (2015). Scolytinae y Platypodinae (Coleoptera: Curculionidae) asociados a manglares de Tabasco, México. Rev. Colomb. Entomol..

[17] Gerónimo-Torres J.D.C., Pérez-de La Cruz M., de La Cruz-Pérez A., Arias-Rodríguez L., Burelo-Ramos C.M. (2021). Diversidad y distribución vertical de escarabajos barrenadores (Coleoptera: Bostrichidae, Curculionidae: Scolytinae, Platypodinae) en un manglar en Tabasco, México. Caldasia.

[18] Lázaro-Dzul M.O., Vargas-Madriz H., Monteòn-Ojeda A., Atkinson T.H., Azuara-Domínguez A. (2024). New distribution records and host plants of two species of *Hypothenemus* (Coleoptera: Curculionidae: Scolytinae) in mangrove ecosystems of Tamaulipas, Mexico. Fla. Entomol..

[19] Lázaro Dzul M.O., Vargas-Madriz H., Azuara-Dominguez A., Vargas-Tovar J.A. (2026). Scolytinae y Platypodinae (Coleoptera: Curculionidae) asociados al ecosistema mangle en Tamaulipas, México. Dugesiana.

[20] da Silva C.O., Trevisan H., de Souza T.S., de Carvalho A.G. (2020). Occurrence of Scolytinae in mangrove with impact trap and in wood of five forest species: Occurrence of insects xylophagous in mangrove. Biosci. J..

[21] Comisión Nacional para el Conocimiento y Uso de la Biodiversidad (CONABIO) La Biodiversidad en Colima: Estudio de Estado. https://www.biodiversidad.gob.mx/region/eeb/estudios/ee_colima.

[22] Atkinson T.H. Bark and Ambrosia Beetles of the Americas. https://www.barkbeetles.info/amer_chklist_overview.php?user_geo=calc_col%3D1&user_geo_title=Colima.

[23] Castrejón-Antonio J.E., Montesinos-Matías R., Acevedo-Reyes N., Tamez-Guerra P., Ayala-Zermeño M.A., Berlanga-Padilla A.M., Arredondo-Bernal H.C. (2017). Especies de *Xyleborus* (Coleoptera: Curculionidae: Scolytinae) asociados a huertos de aguacate en Colima, México. Acta Zool. Mex..

[24] Castrejón-Antonio J.E., Montesinos-Matías R., Tamez-Guerra P., Fuentes Guardiola L.T., Laureano-Ahuelican B., Arredondo-Bernal H.C. (2018). Infestation of *Xyleborus volvulus* (Fabricius) (Coleoptera: Curculionidae: Scolytinae) in *Mangifera indica* L. (Mangifera: Anacardiaceae) in Manzanillo, Colima. Fla. Entomol..

[25] Téllez-García C.P., Valdez-Hernández J.I. (2012). Caracterización Estructural del Manglar en el Estero Palo Verde, Laguna de Cuyutlán, Colima. Rev. Chapingo Ser. Cienc. For. Amb..

[26] Jiménez-Quiroz M.C., González-Orozco F. (1996). Análisis de la estructura del manglar de la laguna de Juluápan, Col., México. Cienc. Pesq..

[27] Hulcr J., Johnson A.J., Gomez D.F. Collecting Bark and Ambrosia Beetles V.2. https://www.protocols.io/view/collecting-bark-and-ambrosia-beetles-5jyl855x7l2w/v2?version_warning=no.

[28] Pérez-Silva M., Equihua-Martínez A., Atkinson T.H., Romero-Nápoles J., López-Buenfil J.A. (2021). Claves ilustradas para la identificación de géneros y especies de la tribu Xyleborini (Curculionidae: Scolytinae) de México. Rev. Mex. Biodivers..

[29] Pérez de la Cruz M., Valdéz Carrasco J.M., Romero Nápoles J., Equihua Martínez A., Sánchez Soto S., de la Cruz Pérez A. (2011). Fluctuación poblacional, plantas huéspedes, distribución y clave para la identificación de Platypodinae (Coleoptera: Curculionidae) asociados al agroecosistema cacao en Tabasco, México. Acta Zool. Mex..

[30] Wood S.L. (2007). Bark and Ambrosia Beetles of South America (Coleoptera: Scolytidae).

[31] Hammer Ø., Harper D.A.T., Ryan P.D. (2001). PAST: Paleontological Statistics Software Package for Education and Data Analysis. Palaeontol. Electron..

[32] Rosas M., Bartorila M., Ocon S. (2016). Laguna del Carpintero, regulador climático en el área urbana de Tampico. Legado Arquit. Diseño.

[33] Fierro-Cabo A., Zamora-Tovar C., González-Medrano F., Vázquez-Lule A.D., Sáenz-Aguilar Y.V., Amador-Cano G., Alcántara-Maya J.A., Rodríguez-Zúñiga M.T. (2022). Caracterización del sitio de manglar GM47 Lomas del Real. Sitios de Manglar con Relevancia Biológica y con Necesidades de Rehabilitación Ecológica.

[34] Rudinsky J.A. (1962). Ecology of Scolytidae. Annu. Rev. Entomol..

[35] Wood S.L. (1986). A reclassification of the genera of Scolytidae (Coleoptera). Gt. Basin Nat. Mem..

[36] Hulcr J., Atkinson T.H., Cognato A.I., Jordal B.H., McKenna D.D., Vega F.E., Hofstetter R.W. (2015). Morphology, Taxonomy, and Phylogenetics of Bark Beetles. Bark Beetles.

[37] Wood S.L., Bright D.E. (1992). A Catalog of Scolytidae and Platypodidae (Coleoptera), Part 2. Taxonomic Index. Gt. Basin Nat. Mem..

[38] Ranger C.M., Reding M.E., Persad A.B., Herms D.A. (2010). Ability of stress-related volatiles to attract and induce attacks by *Xylosandrus germanus* and other ambrosia beetles. Agric. For. Entomol..

[39] Biedermann P.H., Vega F.E. (2020). Ecology and Evolution of Insect–Fungus Mutualisms. Annu. Rev. Entomol..

